# Species‐Specific Controls on Rhizosphere Soil Respiration in a Tropical Deciduous Forest of Bangladesh

**DOI:** 10.1002/pei3.70200

**Published:** 2026-08-09

**Authors:** Mohammad Ashraful Alam, Tahamid Chowdhury Rasif, Liza Kamal, M Azizar Rahaman, Mahim Md. Muntashir Pramanik, Imran Ahmed, Md. Faruque Hossain, Ashfaque Ahmed

**Affiliations:** ^1^ Ecology, Environment and Natural Resource Laboratory, Department of Botany University of Dhaka Dhaka Bangladesh; ^2^ Applied Statistics and Data Science, Institute of Statistical Research and Training University of Dhaka Dhaka Bangladesh; ^3^ Wildlife and Nature Conservation Circle Bangladesh Forest Department Ban Bhaban, Agargaon, Dhaka Bangladesh; ^4^ American International University‐Bangladesh Dhaka Bangladesh

**Keywords:** belowground carbon dynamics, deciduous forest, forest carbon cycling, rhizosphere processes, soil organic carbon, soil respiration

## Abstract

Soil respiration (Rs) is a major pathway of CO_2_ release from terrestrial ecosystems and plays a fundamental role in regulating forest carbon cycling. However, species‐specific controls on rhizosphere soil respiration in tropical deciduous forests remain poorly understood, particularly under tree species with contrasting functional characteristics. This study investigated rhizosphere Rs and associated soil physicochemical properties in the rhizosphere soils of three dominant tree species, *Shorea robusta* , *Albizia procera* , and *Acacia auriculiformis* , in Bhawal National Park, Bangladesh. Rhizosphere soils were collected from two depths (0–15 and 15–30 cm), and soil physicochemical properties were analyzed alongside laboratory‐based measurements of Rs. Soil respiration differed significantly among tree species and soil depths, with the highest values observed in *A. auriculiformis* , followed by *S. robusta* , whereas the lowest in *A. procera* . In contrast, soil organic carbon and total nitrogen were greatest in the rhizosphere of *A. procera* and lowest in *A. auriculiformis* . Multivariate analyses revealed distinct differences in rhizosphere soil characteristics among tree species, primarily associated with soil pH, moisture content, calcium, and organic carbon. Multiple regression analysis identified soil moisture and calcium as the strongest predictors of Rs, explaining 52% of the observed variation. The negative relationship between Rs and soil organic carbon suggests differences in carbon turnover and stabilization rather than carbon quantity alone. Although the underlying mechanisms were not directly examined, the observed differences among species are likely associated with species‐specific belowground functional traits that influence rhizosphere processes. These findings demonstrate that tree species identity is an important determinant of belowground carbon dynamics and highlight the importance of considering species‐specific effects when designing forest restoration and management strategies aimed at maintaining soil carbon storage and ecosystem functioning in tropical deciduous forests.

## 1 Introduction

Forests play a fundamental role in the global carbon cycle by functioning simultaneously as major carbon sinks and sources, thereby regulating atmospheric carbon dioxide (CO_2_) concentrations and influencing climate change (Hurteau 2021; Chaturvedi et al. 2025). Although aboveground biomass has traditionally received considerable attention in forest carbon assessments, belowground processes, particularly soil respiration (Rs), represent one of the largest carbon fluxes from terrestrial ecosystems to the atmosphere (Bond‐Lamberty et al. 2018; Sivaranjani et al. 2025). Soil respiration, defined as the release of CO_2_ from the soil surface, comprises autotrophic respiration from plant roots and heterotrophic respiration associated with microbial decomposition of organic matter and soil fauna activity (Phillips et al. 2017; Alam et al. 2024; Lin et al. 2025). As a major pathway linking soil organic carbon turnover with atmospheric CO_2_ exchange, soil respiration is widely recognized as a key indicator of ecosystem carbon cycling and climate feedbacks (Guo et al. 2023; Bond‐Lamberty et al. 2024).

Soil respiration is regulated by a complex interaction of environmental and biological factors that influence root metabolism, microbial processes, and substrate availability (Kruse et al. 2013; Lin et al. 2025). Among these, soil moisture is widely regarded as one of the dominant controls because it regulates oxygen diffusion, microbial activity, and substrate transport, whereas both drought and excessive water saturation suppress respiratory activity (Xu et al. 2022). Soil pH influences nutrient availability and microbial community structure, while soil organic carbon provides the principal substrate supporting biological respiration (Guo et al. 2023; Bond‐Lamberty et al. 2024; Xia et al. 2024; Sharma et al. 2025). Essential nutrients, including nitrogen, phosphorus, potassium, calcium, and magnesium, also contribute to soil respiration by regulating plant nutrition, enzyme activity, and microbial growth (Fu et al. 2021; Sun et al. 2025). However, the influence of these soil properties is often species dependent because different tree species modify rhizosphere environments through their effects on litter inputs, root traits, nutrient acquisition strategies, and belowground carbon allocation.

The rhizosphere is one of the most biologically active compartments of forest ecosystems, where roots, microorganisms, and soil continuously interact through the exchange of carbon, nutrients, and signaling compounds. Root exudates and other rhizodeposits provide readily available carbon substrates that support microbial metabolism and contribute substantially to soil respiration (Velmourougane et al. 2017; Das et al. 2022). At the same time, differences in root morphology, rhizosphere chemistry, nutrient uptake strategies, and litter characteristics among tree species create distinct soil environments that influence carbon turnover and nutrient cycling (Moreno Roblero et al. 2020; Jin et al. 2023). Consequently, tree species identity has emerged as an important determinant of belowground ecosystem processes and soil carbon dynamics.

Tropical forests contribute substantially to global terrestrial carbon cycling because warm temperatures and favorable moisture conditions promote high biological activity and rapid organic matter turnover (Lal 2005; Gupta et al. 2023). Nevertheless, tropical deciduous forests remain comparatively understudied with respect to rhizosphere soil respiration, particularly in South Asia, where information on the relationships between soil respiration and soil physicochemical properties across dominant tree species is still limited.

Bangladesh's tropical deciduous forests have undergone extensive ecological change due to deforestation, fragmentation, urban expansion, and plantation‐based restoration programmes (Mondol et al. 2010; Rahman et al. 2023). Bhawal National Park (BNP), one of the largest remaining tracts of tropical deciduous forest in Bangladesh, exemplifies these changes (Kabir and Ahmed 2005; Alauddin et al. 2020). Historically dominated by *Shorea robusta* , the forest now also contains extensive stands of *Albizia procera* and plantation‐grown *Acacia auriculiformis* (Rahman et al. 2009; Mukul et al. 2021). These contrasting tree species differ markedly in growth characteristics, nutrient cycling, litter production, and rhizosphere interactions, making BNP an ideal natural system for investigating species‐specific variation in belowground carbon dynamics.

Previous investigations in Bhawal National Park have primarily examined land‐use change, forest degradation, and soil organic carbon stocks (Mondol et al. 2010; Propa et al. 2021; Rahman et al. 2023). However, little information is available regarding how different dominant tree species influence rhizosphere soil respiration through changes in soil physicochemical properties. In particular, few studies have simultaneously evaluated soil respiration together with multiple soil properties to identify the principal environmental drivers of rhizosphere carbon dynamics in tropical deciduous forests. Addressing this knowledge gap is important for understanding belowground ecosystem functioning and supporting evidence‐based forest restoration and management.

Understanding species‐specific regulation of soil respiration is particularly relevant because differences among tree species may influence soil carbon turnover, nutrient cycling, and long‐term soil carbon storage. Although previous studies have shown that some plantation or introduced species can modify soil properties and belowground processes, these responses vary considerably among species and environmental conditions. Therefore, species‐specific ecological characteristics, rather than native or introduced status alone, are likely to determine their influence on rhizosphere soil processes.

Against this background, the present study investigated rhizosphere soil respiration and the physicochemical properties of rhizosphere soils associated with three dominant tree species ( *Shorea robusta* , *Albizia procera* , and *Acacia auriculiformis* ) in Bhawal National Park, Bangladesh. Specifically, the objectives were to (i) quantify soil respiration and associated soil physicochemical properties across tree species and soil depths, (ii) identify the major environmental factors regulating soil respiration using multivariate and regression analyses, and (iii) evaluate species‐specific differences in rhizosphere carbon dynamics. We hypothesized that soil respiration differs among tree species because species‐specific functional traits influence rhizosphere soil conditions, particularly soil moisture, organic carbon, and nutrient availability. By integrating soil respiration measurements with comprehensive soil physicochemical analyses, this study provides new insights into species‐specific controls on belowground carbon dynamics and contributes scientific evidence for sustainable forest restoration and management in tropical deciduous forests.

## 2 Materials and Methods

### 2.1 Study Area

Bhawal National Park (BNP), locally known as the Rajendrapur Gajari (Sal) Forest, is located in Gazipur district, approximately 40 km north of Dhaka, Bangladesh. Geographically, it lies between latitudes 24°01′‐24°13′ N and longitudes 90°14′‐90°30′ E, at an altitude of 10–15 m above mean sea level (Kabir and Ahmed 2005; Islam et al. 2017). Established in 1974 and declared a national park in 1982 under the Bangladesh Wildlife Act, BNP spans 5022 ha, including a 950‐ha core area, and is categorized under IUCN Management Category IV (Kabir and Ahmed 2005; Alauddin et al. 2020).

The Park features gently undulating low upland areas called “Chalas,” interspersed with shallow depressions known as “Baids,” creating micro‐topographical variation that influences hydrology and vegetation distribution. The climate is moderate, with an average annual temperature of 26°C, humidity of 72%, and mean annual precipitation of 2204 mm. The soil is characteristically acidic, affecting nutrient availability and ecosystem dynamics (Alauddin et al. 2020). Although native Sal trees ( *Shorea robusta* ) were extensively removed, conservation programs have restored vegetation, with exotic trees now covering 90% of the park. BNP hosts 221 plant species, including 43 trees, 24 climbers, and 105 herbs (Islam et al. 2017). Despite its ecological significance, the park faces severe pressure from urbanization and over 150 industries in the vicinity, leading to habitat degradation (Alauddin et al. 2020).

### 2.2 Soil Sampling and Analytical Procedures

**FIGURE 1:**
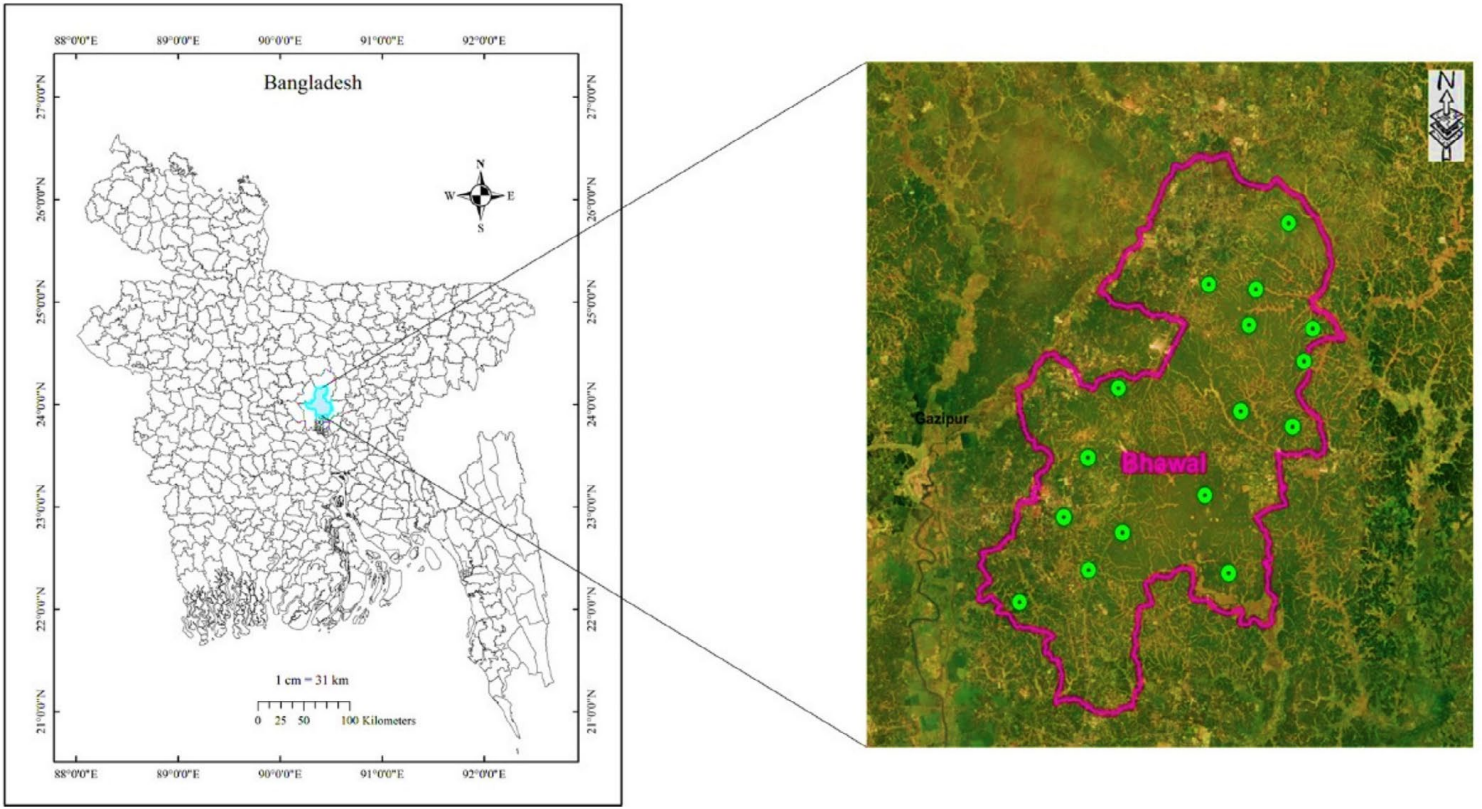
Map representing the location of the study area within Bhawal National Park, Bangladesh.

In August 2024, rhizosphere soil samples were collected from Bhawal National Park using a stratified sampling approach based on the dominant tree species. Sample plots (10 × 10 m) were established within relatively homogeneous stands dominated by one of the three target species ( *Shorea robusta* , *Albizia procera* , or *Acacia auriculiformis* ) (Figure 1). Because *S. robusta* is the naturally dominant tree species in Bhawal National Park, a larger number of suitable stands was available for sampling. In contrast, naturally occurring stands dominated by *A. procera* and plantation stands dominated by *A. auriculiformis* were comparatively limited within the study area. Consequently, ten plots were established under *S. robusta* and three plots each under *A. procera* and *A. auriculiformis* . This sampling design reflects the natural distribution and availability of dominant stands within the forest rather than an intentional imbalance in experimental replication.

Within each plot, rhizosphere soil samples were collected from two depths (0–15 cm and 15–30 cm) using a soil auger and core sampler. Rhizosphere soil was separated immediately after sampling by gently shaking the roots to remove loosely attached soil, after which the soil adhering directly to the roots was carefully collected using sterilized forceps. The samples were placed in airtight polyethylene bags, transported to the Ecology, Environment and Natural Resource Laboratory, and stored under appropriate conditions until physicochemical analyses were performed. The soil physicochemical properties, including pH, conductivity, MC, soil texture, TN, TP (Okalebo et al. 1993), OC (Walkley and Black 1934), exchangeable Ca, exchangeable Mg (Huq and Alam 2005), and available K (Allen et al. 1974), were analyzed using standard laboratory methods.

### 2.3 Soil Respiration

Soil respiration rate was determined by measuring CO_2_ evolution through its conversion to CO_3_^2−^ in the presence of excess OH^−^, with the consumed OH^−^ calculated via chemical titration (Haney et al. 2008). Each milliequivalent of OH^−^ consumed corresponds to 2 millimoles of substrate carbon oxidized or 6.00 mg of carbon. For the analysis, 25 g of oven‐dry equivalent soil (adjusted for initial moisture) was transferred to one side of a Mason jar, and distilled water was added to achieve 30% moisture by weight. A labeled Falcon tube containing 20 mL of 1 N NaOH was carefully placed inside each jar to avoid spillage that could harm handlers or soil microorganisms. The jars were tightly sealed and incubated for a week to prevent any CO_2_ exchange with the atmosphere. After incubation, Falcon tubes were removed, and 10 mL of 2 N BaCl_2_ was added to each tube to precipitate BaCO_3_. Solutions were centrifuged, and 10 mL of the supernatant from each tube was pipetted into labeled Erlenmeyer flasks. After adding 40 mL of distilled water and phenolphthalein indicator, the solution was titrated with 0.25 N HCl to a colorless endpoint. The volume of HCl used was recorded, and the amount of consumed OH^−^ was calculated to quantify the oxidized substrate carbon.

### 2.4 Statistical Analysis

All statistical analyses were conducted using R Studio (version 4.4.1). Data normality was assessed using the Shapiro–Wilk test. Analysis of Variance (ANOVA) was employed to evaluate the effects of different plant species, soil depths, and their interactions on soil physicochemical properties. Tukey's post hoc test was conducted to determine significant differences among groups, where groups sharing the same letter are not significantly different from each other (*p* < 0.05). Pearson's correlation analysis was performed to examine relationships among the studied parameters. Additionally, multivariate analyses, including Principal Component Analysis (PCA), Cluster analysis, Canonical Discriminant Analysis (CDA), and Multiple regression analysis, were conducted to identify patterns, groupings, and relationships within the data.

## 3 Results

### 3.1 Soil Physicochemical Properties

The soil physicochemical properties of rhizosphere soil of three dominant plant species of BNP are described in Table 1. The rhizosphere soil pH values differed significantly among the three species (*F* = 37.46, *p <* 0.001) (Tables S1 and S2), with *A. auriculiformis* showing the highest pH (5.29 ± 0.18), followed by *S. robusta* (4.82 ± 0.11) and *A. procera* (4.81 ± 0.11).

**TABLE 1:** Soil physicochemical properties of rhizosphere soil of three dominant plant species of Bhawal National Park (BNP).

Soil properties	*Acacia auriculiformis*	*Albizia procera*	*Shorea robusta*
pH	5.29 ± 0.18 (a)	4.81 ± 0.11 (b)	4.82 ± 0.11 (b)
MC (%)	24.02 ± 1.29 (ab)	21.82 ± 1.27 (b)	24.90 ± 2.34 (a)
OC (%)	0.49 ± 0.19 (b)	0.90 ± 0.25 (a)	0.67 ± 0.10 (b)
Conductivity (μS/cm)	119.03 ± 10.46 (a)	128.33 ± 35.01 (a)	131.13 ± 33.03 (a)
TN (%)	0.06 ± 0.01 (c)	0.13 ± 0.03 (a)	0.10 ± 0.02 (b)
TP (%)	0.10 ± 0.02 (a)	0.10 ± 0.02 (a)	0.11 ± 0.03 (a)
Sand (%)	47.83 ± 4.45 (a)	39.89 ± 3.26 (b)	43.98 ± 5.66 (ab)
Silt (%)	27.17 ± 3.82 (a)	20.60 ± 2.73 (b)	19.28 ± 2.5 (b)
Clay (%)	25 ± 7.56 (b)	39.64 ± 2.00 (a)	37.00 ± 4.67 (a)
Ca (meq. /100 g)	4.80 ± 0.13 (a)	4.23 ± 0.35 (b)	4.40 ± 0.11 (b)
Mg (meq/100 g)	1.34 ± 0.05 (a)	1.19 ± 0.09 (c)	1.27 ± 0.04 (b)
K (ppm)	66.83 ± 13.61 (a)	74.27 ± 20.30 (a)	71.58 ± 18.26 (a)

*Note:* Values are mean ± standard deviation. Groups sharing the same letter within a row are not significantly different (*p* < 0.05, Tukey's HSD test). Exchangeable Ca and Mg are expressed as meq 100/g soil, whereas available K is expressed as ppm, according to the analytical methods described in the Materials and Methods.

Abbreviations: Ca, Calcium; EC, Electrical Conductivity; K, Potassium; MC, Moisture Content; Mg, Magnesium; OC, Organic Carbon; TN, Total Nitrogen; TP, Total Phosphorus.

**FIGURE 2:**
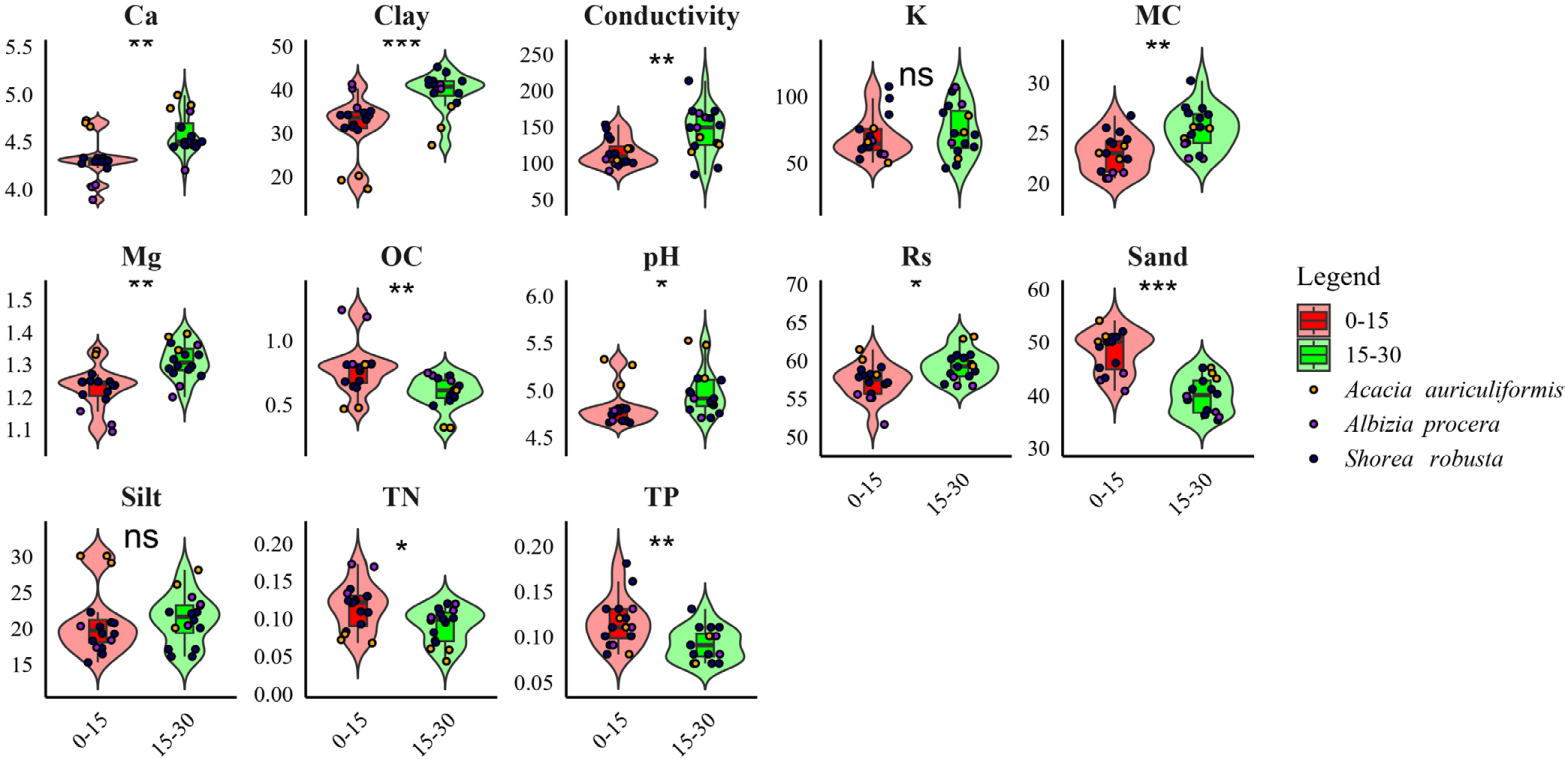
Results of Wilcoxon tests comparing the distribution of soil properties across different soil depths, displayed as a violin‐box plot. The black horizontal line represents the median, and the width of the violin plot corresponds to the distribution of the data, indicating kernel probability density. Different color‐coded jitter points represent different plant species. Ca, Calcium; EC, Electrical Conductivity; K, Potassium; MC, Moisture Content; Mg, Magnesium; NS, Not Significant; OC, Organic Carbon; TN, Total Nitrogen; TP, Total Phosphorus. *p* < 0.05 = *, *p* < 0.01 = **, *p* < 0.001 = ***.

Moisture content differed significantly among the rhizosphere soils of the three plant species (*F* = 7.41, *p* < 0.01) (Table S2), with relatively similar MC for *A. auriculiformis* (24.02% ± 1.29%) and *S. robusta* (24.90% ± 2.34%), and lower for *A. procera* (21.82% ± 1.27%) and moisture content significantly increased with soil depth (*p <* 0.01) (Table 1 and Figure 2).

The rhizosphere soils of *A. procera* contained significantly higher OC concentration (0.90% ± 0.25%) and TN (0.13% ± 0.03%) compared to the other species. *S. robusta* and *A. auriculiformis* followed with moderate to low values of OC (0.67% ± 0.10%, 0.49% ± 0.19%) and TN (0.10% ± 0.02%, 0.06% ± 0.01%), respectively (Table 1). In contrast, total phosphorus did not differ significantly among species (*F* = 0.69, *p >* 0.05) (Table S2), with values ranging between 0.10% ± 0.02% and 0.11% ± 0.03%.

There were significant differences in the rhizosphere soil texture among the three plant species (Table S2). The rhizosphere soil of *A. auriculiformis* , characterized by the highest sand content (47.83% ± 4.45%) and moderate levels of silt (27.17% ± 3.82%) and clay (25% ± 7.56%), was classified as sandy clay loam. While the rhizosphere soil of *A. procera* (39.89% ± 3.26% sand, 39.64% ± 2.00% clay and 20.60% ± 2.73% silt) and the rhizosphere soil of *S. robusta* (43.98% ± 5.66% sand, 37.00% ± 4.67% clay and 19.28% ± 2.50% silt) were both classified as clay loam due to their higher clay content and moderate sand and silt proportions (Table 1). For all the species, the sand content of soil decreased and the clay content increased with increasing soil depth (Table S1 and Figure 2).

Electrical conductivity showed no significant differences among species (*F* = 0.612, *p >* 0.05) (Table S2), with values between 119.03 ± 10.46 μS/cm ( *A. auriculiformis* ) and 131.13 ± 33.03 μS/cm ( *S. robusta* ). Similarly, potassium did not vary significantly among species (*F* = 0.738, *p >* 0.05) (Table S2), with values ranging from 66.83 ± 13.61 ppm ( *A. auriculiformis* ) to 74.27 ± 20.30 ppm ( *A. procera* ). In contrast, significant differences were observed for Ca and Mg, with the highest Ca (4.80 ± 0.13 meq/100 g) and Mg (1.34 ± 0.05 meq/100 g) in *A. auriculiformis* . All soil physicochemical properties, except silt and K, differed significantly between the two depths (Figure 2). Plant species and soil depth exhibited a significant interaction effect on calcium, clay, and silt (Table S2).

### 3.2 Soil Respiration, Plant Species, and Depth

**FIGURE 3:**
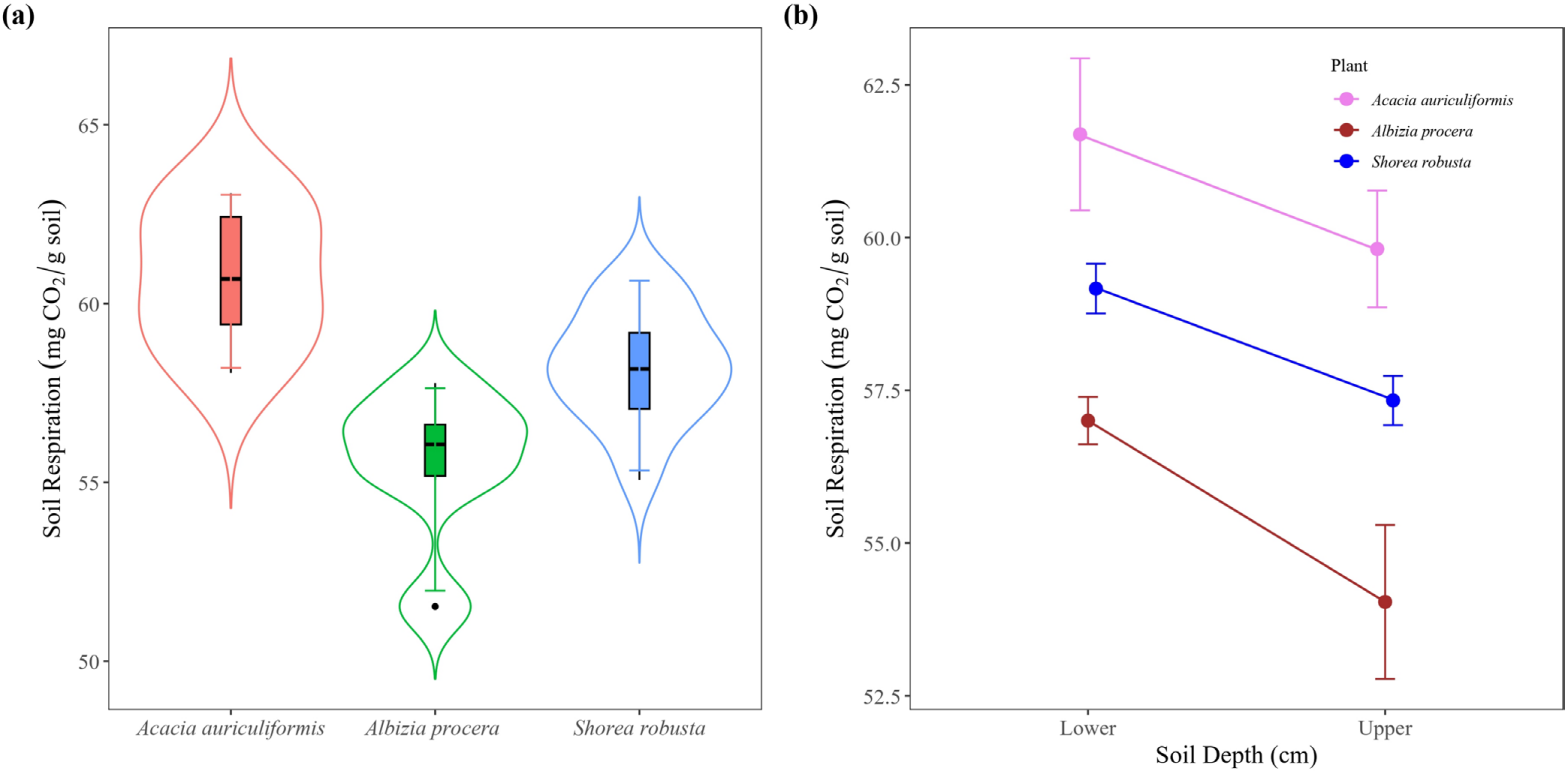
(a) Soil respiration (Rs) (mg CO_2_/g soil) of three dominant plant species, displayed as a violin‐box plot, where the black horizontal line and dot represent the median and outlier, respectively. The width of the violin plot corresponds to the distribution of the data, indicating kernel probability density. Groups sharing the same letter are not significantly different from each other. (b) Average Rs (mg CO_2_/g soil) of rhizosphere soil from those plants at two different soil depths.

The rhizosphere Rs varied significantly among three dominant plants of the studied forest (Table S2). Tukey post hoc test showed that Rs of each plant differed significantly from each other, while the highest Rs was found in the rhizosphere soil of *A. auriculiformis* (60.75 ± 2.00 mg CO_2_/g soil), followed by *S. robusta* (58.25 ± 1.56 mg CO_2_/g soil) and the lowest Rs was found in the rhizosphere of *A. procera* (55 ± 2.18 mg CO_2_/g soil) (Figure 3a). Contrary to OC, rhizosphere Rs showed significant increase with increasing depth for each plant (Figures 2 and 3b). Similar to OC both plant species and soil depth independently influenced Rs, but their interaction remained statistically insignificant (Table S2).

### 3.3 Multivariate Analysis of Species‐Specific Soil Properties

**FIGURE 4:**
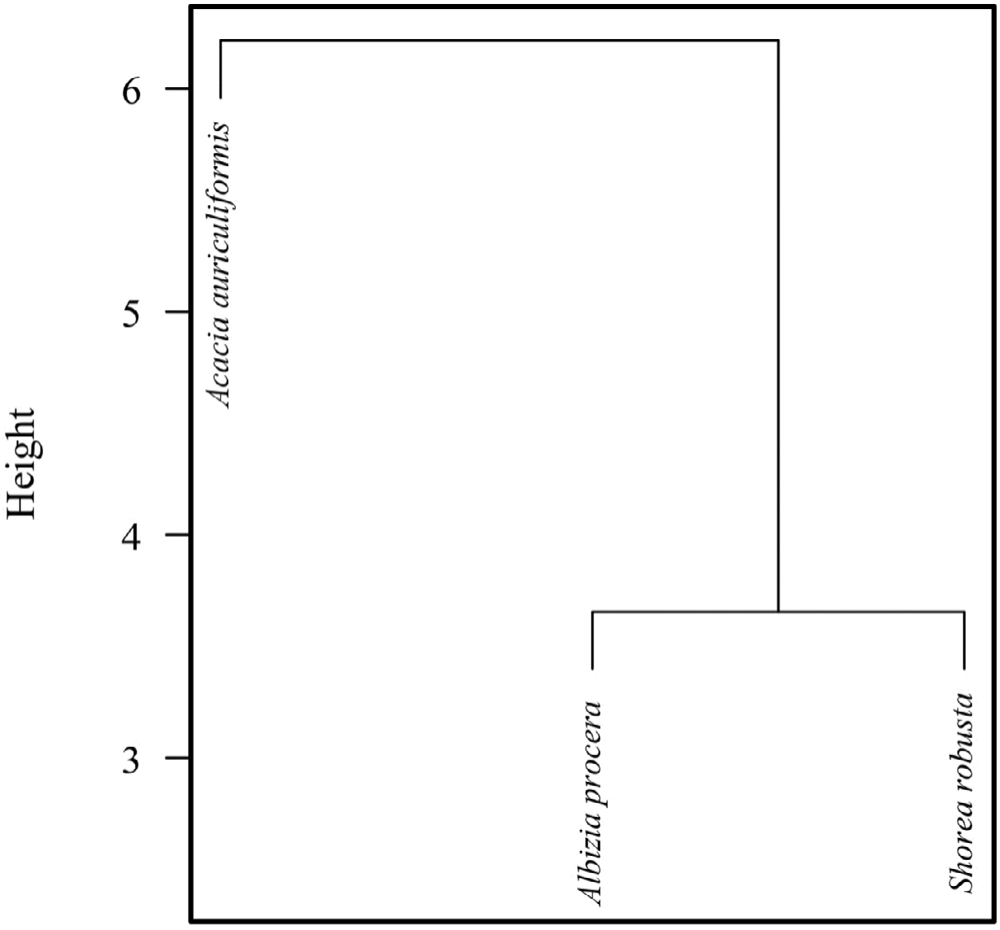
Cluster analysis of plant species based on soil physicochemical properties and soil respiration (Rs).

In the cluster analysis, two distinct groups emerged: one comprising *A. auriculiformis* and the other combining *S. robusta* and *A. procera* (Figure 4). This grouping was further supported by CDA, where *A. auriculiformis* was primarily separated along the first canonical axis (Can1), which accounted for 86.7% of the variation, mainly due to its association with soils characterized by higher pH, greater silt content, and elevated calcium levels (Figure S1). In contrast, *S. robusta* and *A. procera* shared a preference for similar soil conditions with higher OC, TN, and MC, which contributed to a closer association on Can2, explaining 13.3% of the variation. MANOVA revealed that plant species had a significant effect on the variation of soil properties, supported by all four tests including Pillai's Trace (*F* = 7.31, *p* < 0.001), Wilks' Lambda (*F* = 8.52, *p* < 0.001), Hotelling‐Lawley Trace (*F* = 9.85, *p* < 0.001), and Roy's Largest Root (*F* = 18.24, *p* < 0.001) (Table 2), and PERMANOVA also supported the cluster analysis, as no significant differences were found between *S. robusta* and *A. procera* (*p* = 0.276), whereas significant variations existed between *S. robusta* and *A. auriculiformis* (*p* = 0.015) and between *A. procera* and *A. auriculiformis* (*p* = 0.004) (Table 3).

**TABLE 2:** MANOVA for rhizosphere soil physicochemical properties of three plant species of BNP.

Test	Df	Statistic	Approx. F	Num Df	Den Df	*p*	Significance
Pillai's Trace	2	1.7453	7.3078	30	32	1.175e‐07	***
Wilks' Lambda	2	0.011	8.5247	30	30	3.791e‐08	***
Hotelling‐Lawley Trace	2	21.11	9.8515	30	28	1.636e‐08	***
Roy's Largest Root	2	17.097	18.237	15	16	2.782e‐07	***

Abbreviations: Den Df, Denominator Degrees of Freedom; NS, Not Significant; Num Df, Numerator Degrees of Freedom.

*** *p* < 0.001.

**TABLE 3:** Results of PERMANOVA analysis comparing soil physicochemical properties across the rhizosphere soils of three tree species: *Shorea robusta, Albizia procera, an*d *Acacia auriculiformis* .

Comparison	Df	SS	MS	F Model	*R* ^2^	*p*	Significance
*Shorea robusta* versus *Albizia procera*	1	0.0071	0.0071	1.216	0.04822	0.276	NS
*Shorea robusta* versus *Acacia auriculiformis*	1	0.0194	0.0194	3.856	0.13843	0.015	*
*Albizia procera* versus *Acacia auriculiformis*	1	0.0243	0.0243	5.227	0.34326	0.004	**

*Note:* The test was performed with 999 permutations, and significance levels are indicated as *NS =* Not significant, *p < 0.05, and **p < 0.01.

### 3.4 Identification of Key Soil Properties Influencing Soil Respiration

**FIGURE 5:**
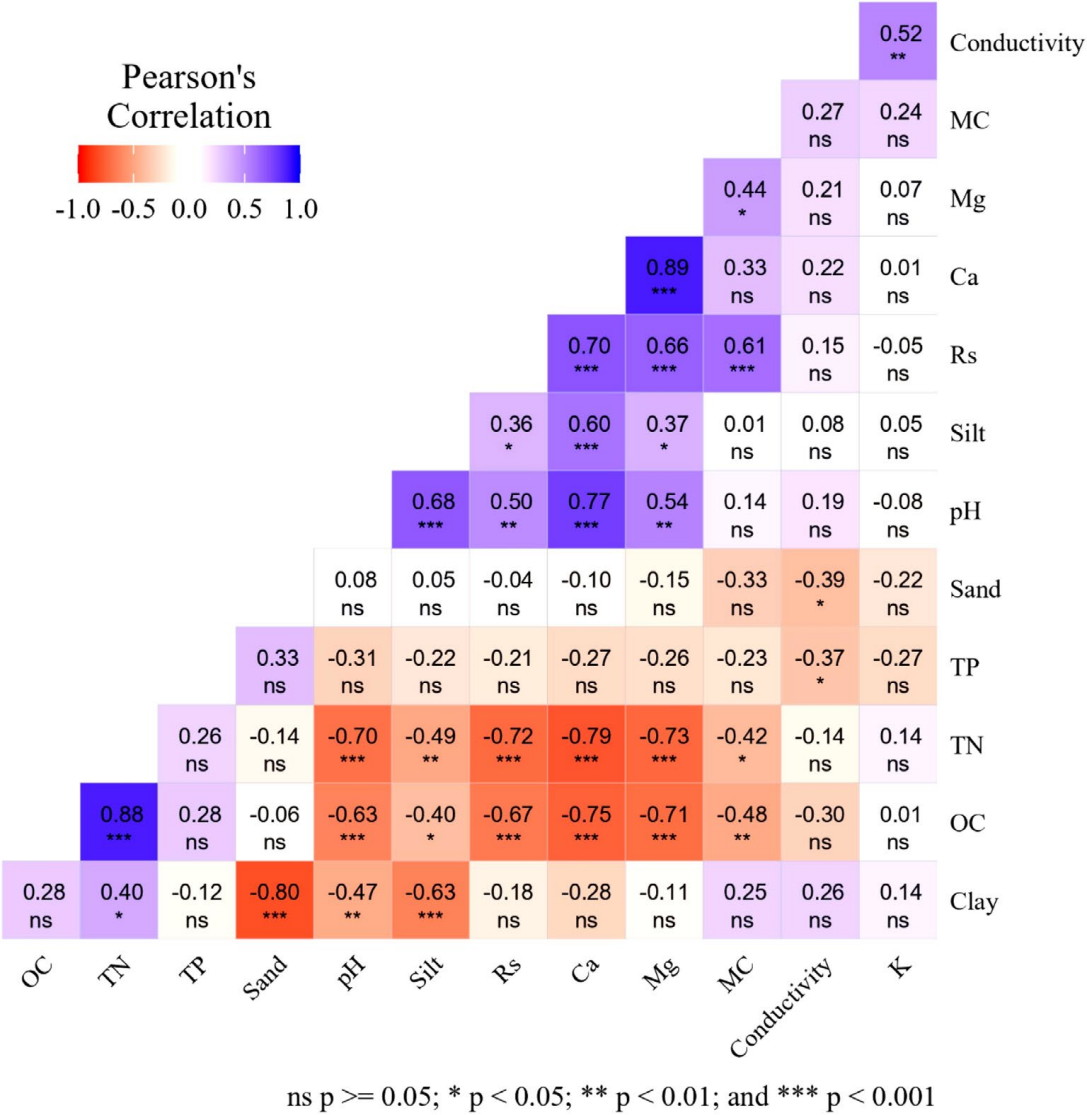
Correlation matrix among soil respiration (Rs) and soil physicochemical properties. Red and violet colors indicate positive and negative correlations, respectively. The soil physicochemical properties include pH (soil pH), moisture content (MC), conductivity, organic carbon (OC), total nitrogen (TN), total phosphorus (TP), and the concentrations of Ca, Mg, K, sand, silt, and clay.

To identify the soil properties influencing Rs, Pearson correlation analysis was first performed (Figure 5). Rs showed significant positive correlations with pH, silt content, MC, Ca, and Mg, while negative correlations were observed with OC and TN (*p* < 0.05). However, strong intercorrelations among these variables indicated multicollinearity, limiting their direct use in multiple linear regression.

**FIGURE 6:**
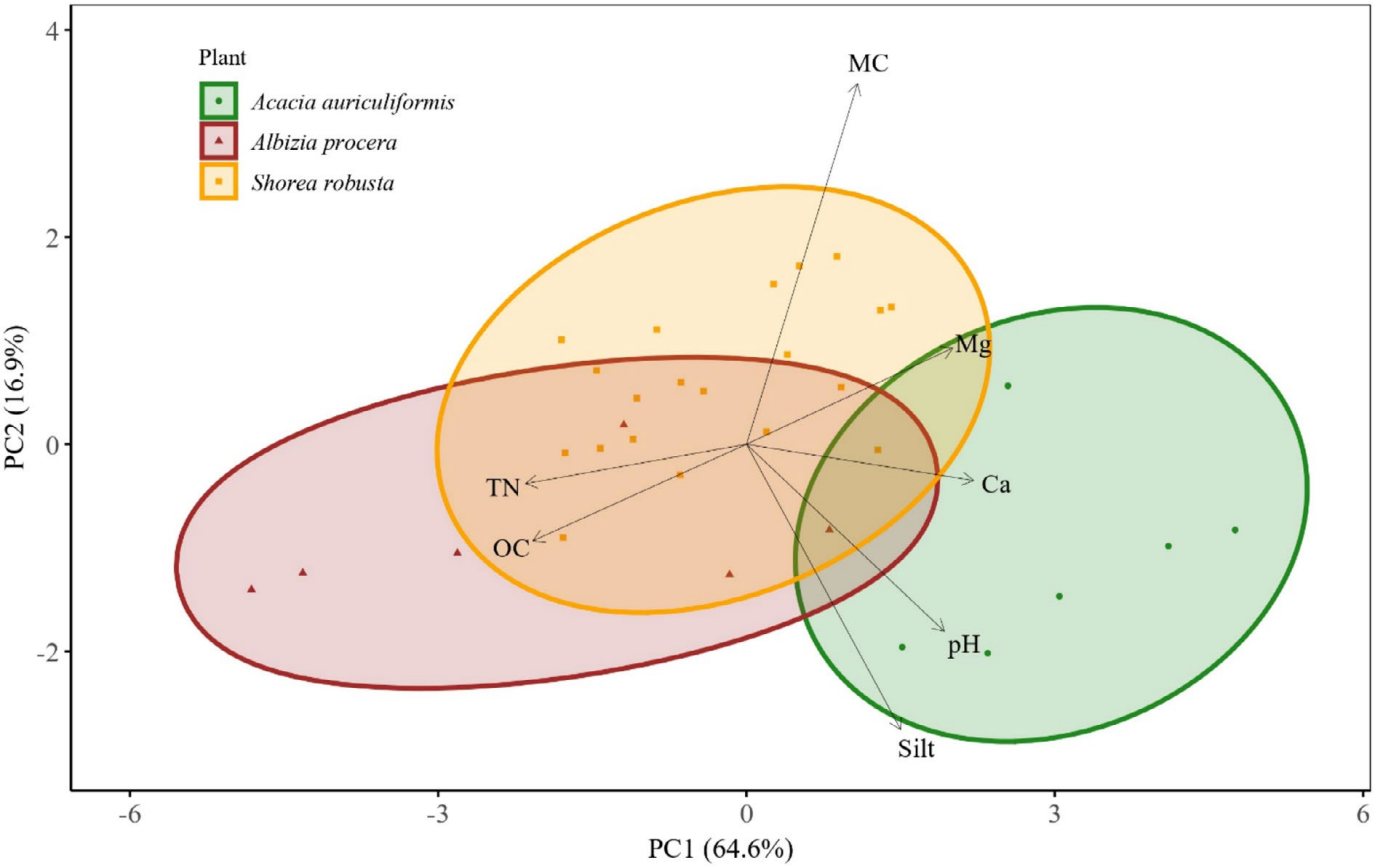
Principal component analysis (PCA) biplot of soil physicochemical properties correlated with soil respiration (Rs), used to identify predictors of Rs prior to multiple regression analysis. Data points are color‐coded to represent three plant species. The soil physicochemical properties include pH (soil pH), OC (organic carbon concentration), MC (moisture content), TN (total nitrogen), and the concentrations of Ca, Mg, and silt.

To address this, PCA was conducted on the eight soil properties significantly correlated with Rs. The first two principal components (PC1 and PC2) accounted for 64.6% and 16.9% of the total variance, respectively (Figure 6 and Table S3). PC1 was primarily influenced by Mg, Ca, OC, TN, and SOC, while PC2 was dominated by pH, MC, and silt, each with high loading values (> 0.35). Due to multicollinearity within PC1, only Ca, which had the highest loading, was selected for further analysis. The variables from PC2 (pH, MC, and silt) were not affected by multicollinearity and were all retained.

Subset regression analysis was then performed using Ca, pH, MC, and silt to determine the most effective model for explaining Rs. Among the tested models, the combination of MC and Ca provided the best predictive performance, based on R^2^, adjusted R^2^, and the lowest C(p), AIC, SBC, and SBIC values (Table 4).

**TABLE 4:** Subsets regression summary for model selection.

Model	*R* ^2^	Adjusted *R* ^2^	C(p)	AIC	SBIC	SBC
Ca	0.4894	0.4724	11.40	129.150	37.52	133.547
MC and Ca	**0.6494**	**0.6252**	**1.058**	**119.126**	**29.34**	**124.989**
MC, Ca, and Silt	0.65	0.6125	3.009	121.068	31.669	128.397
MC, Ca, Silt, and pH	0.65	0.5983	5.000	123.057	34.028	131.852

*Note:* Bold values indicate that the combination of MC and Ca provided the best predictive performance, based on R2, adjusted R2, and the lowest C(p), AIC, SBC, and SBIC values. As this is the best predictive model so bold was used here.

The final multiple linear regression model describing the influence of soil properties on Rs under laboratory incubation conditions is:

Rs=24.7132+0.4299MC+5.2066Ca

This model indicates that both moisture content and calcium significantly and positively influence Rs (Table S4).

## 4 Discussion

### 4.1 Species Identity, Functional Traits, and Rhizosphere Functioning

This study demonstrates that tree species identity exerts a strong control over rhizosphere soil properties and soil respiration (Rs) in Bhawal National Park, highlighting the importance of species‐specific belowground processes in tropical deciduous forests. Significant interspecific differences in most soil physicochemical properties and Rs indicate that vegetation type is a key determinant of soil carbon dynamics, consistent with observations from other deciduous forest ecosystems (Si et al. 2018; Sarkar et al. 2024). These differences are likely associated with species‐specific functional traits, including variations in root morphology, litter characteristics, exudation patterns, and other rhizosphere processes that influence nutrient cycling and carbon turnover (Gan et al. 2021; Wang et al. 2024).

*Acacia auriculiformis* exhibited a distinct soil environment compared to the native species *Shorea robusta* and *Albizia procera* , as reflected by higher soil pH, calcium and magnesium concentrations, and elevated Rs rates. Such differentiation aligns with previous studies reporting that fast‐growing species with high biomass production often modify soil structure, nutrient availability, and rhizosphere conditions more intensively than slower‐growing native species (Binkley and Giardina 1998; Lal 2005; Jaafar and Sukri 2023). The extensive root system and rapid growth strategy of *A. auriculiformis* may enhance rhizosphere microbial stimulation, leading to accelerated SOC mineralization and higher CO_2_ emissions from soil (Lee and Woo 2012; Ontong et al. 2023).

Interestingly, however, not all fast‐growing species exhibit similar effects. The contrasting responses of *A. procera* and *A. auriculiformis* , both Fabaceae, suggest species‐specific functional traits, not origin, drive belowground carbon dynamics. Despite both fixing nitrogen, *A. procera* exhibited lower Rs and higher SOC, likely due to differences in litter quality, root turnover, or mycorrhizal associations (Binkley and Giardina 1998; Forrester et al. 2006).

The clustering of *S. robusta* and *A. procera* in multivariate analyses suggests comparable belowground functioning between these two native species. They appear better adapted to local soil conditions, maintaining higher SOC and TN, which supports long‐term carbon storage rather than rapid turnover (Lone et al. 2019). The separation between species in cluster and discriminant analyses highlights the ecological consequences of introducing non‐native species into tropical forest systems, particularly with respect to soil carbon stability, while also reinforcing the importance of understanding species‐specific functional traits.

### 4.2 Soil Physicochemical Properties and Depth Effects

The acidic soil conditions observed across all species are typical of tropical deciduous forests and reflect organic acid production, intense weathering, and cation leaching under high rainfall regimes (Wuyts et al. 2013; Khanikar et al. 2023). The relatively higher pH in the rhizosphere of *A. auriculiformis* may be linked to altered nitrification‐ammonification dynamics and increased base cation availability, particularly calcium and magnesium (Binkley and Giardina 1998; Jaafar et al. 2022). Such shifts in pH can influence microbial community structure and enzymatic activity, indirectly affecting soil respiration.

Surface soils contained higher SOC and TN compared to deeper layers, consistent with greater litter input, root density, and microbial activity near the soil surface (Jobbágy and Jackson 2001; Qi et al. 2021). However, SOC concentrations were lowest under *A. auriculiformis* , suggesting reduced carbon retention despite higher respiration rates. This pattern may reflect faster organic matter turnover driven by enhanced microbial activity, combined with the lower quality or slower decomposition of *A. auriculiformis* litter reported in previous studies (Mondol et al. 2010; Jaafar et al. 2022).

Soil texture further modulated carbon dynamics. The higher clay content observed in the rhizosphere soils of *A. procera* likely contributed to greater SOC stabilization through mineral‐organic associations, which protect organic matter from microbial decomposition (Sebastian et al. 2023; Yao et al. 2023). This mechanism may partly explain the lower Rs observed under *A. procera* , despite its higher SOC content. The increase in clay content and moisture with depth also influenced Rs patterns, emphasizing the combined role of physical protection and moisture availability in regulating soil CO_2_ emissions (Hao et al. 2025).

### 4.3 Controls on Soil Respiration

Although several soil properties were correlated with Rs, multivariate and regression analyses identified soil moisture content and calcium as the most influential predictors. Soil moisture regulates microbial metabolism, substrate diffusion, and oxygen availability, making it one of the most consistent drivers of Rs across ecosystems (Xu et al. 2022; Huang et al. 2023). Calcium, meanwhile, plays a crucial role in soil aggregation and stabilization of organic matter‐clay complexes, indirectly enhancing microbial accessibility to substrates while maintaining soil structure (Bronick and Lal 2005; Roychand and Marschner 2015). The positive effect of calcium on Rs observed here suggests that base cation availability can enhance microbial activity under acidic tropical soils (Patra et al. 2021).

Interestingly, SOC and TN were negatively correlated with Rs, contrary to the expectation that higher carbon availability would increase respiration. This pattern likely reflects differences in carbon quality rather than quantity. Microbial communities preferentially decompose labile carbon pools, while recalcitrant carbon fractions contribute to SOC accumulation but are less readily respired (Pisani et al. 2014; Tamura and Suseela 2021). Deeper soils, despite lower SOC concentrations, may contain more labile carbon and higher moisture, supporting increased Rs. This interpretation is consistent with the observed increase in Rs with soil depth.

The regression model explained 52% of the variation in Rs, indicating that additional environmental drivers such as temperature, seasonal rainfall, root phenology, and microbial community composition likely contribute to unexplained variability (Huang et al. 2014).

### 4.4 Implications for Forest Management and Restoration

The findings of this study have important implications for forest management and restoration in tropical deciduous forests. The higher soil respiration observed in rhizosphere soil of *Acacia auriculiformis* compared with *Shorea robusta* and *Albizia procera* indicates greater belowground carbon turnover under this species. Although the mechanisms underlying these differences were not directly investigated, they are likely associated with species‐specific functional characteristics that influence rhizosphere soil conditions and carbon cycling. As soil respiration represents a major pathway of carbon release from forest soils, differences among dominant tree species may influence ecosystem carbon dynamics over time (Bond‐Lamberty et al. 2018; Fan et al. 2024). The contrasting responses of *A. procera* and *A. auriculiformis* , despite both belonging to the Fabaceae, further demonstrate that species‐specific functional traits rather than species origin alone are more likely to regulate rhizosphere carbon dynamics (Binkley and Giardina 1998; Forrester et al. 2006). These findings suggest that species selection for afforestation and restoration should consider both aboveground productivity and belowground ecosystem functioning. Species capable of maintaining relatively greater soil organic carbon while exhibiting lower soil respiration may contribute more effectively to long‐term soil carbon storage. Future studies incorporating litter characteristics, root functional traits, and microbial community composition are needed to clarify the mechanisms underlying these species‐specific differences and to improve sustainable restoration strategies.

### 4.5 Limitations and Future Research Directions

This study was conducted during a single season, limiting insights into temporal variability in soil respiration and soil properties. Seasonal fluctuations in temperature, moisture, and root activity are likely to influence Rs dynamics substantially. Additionally, microbial community composition and functional traits were not assessed, despite their central role in regulating soil carbon processes.

A notable limitation of this study is the absence of litter chemical and biochemical characterization (C:N ratio, lignin content, phenolic compounds), which are known to influence microbial decomposition and soil respiration rates (Gan et al. 2021). Such data would have allowed more robust attribution of species‐specific effects to functional traits rather than origin‐based categories. Future studies should integrate litter quality assessment to better understand the mechanisms underlying species‐specific controls on soil carbon dynamics.

The uneven number of replicate plots among species represents another limitation of this study. Future studies should aim for balanced sampling designs where feasible, particularly when comparing species with contrasting ecological roles.

Future studies should integrate molecular approaches to characterize microbial diversity, quantify labile and recalcitrant carbon fractions, and incorporate additional environmental drivers to better predict soil respiration under different forest management scenarios. Long‐term monitoring across multiple seasons would also help capture temporal dynamics and improve understanding of the mechanisms driving species‐specific effects on soil carbon processes.

## 5 Conclusions

This study demonstrates that tree species identity is an important determinant of rhizosphere soil respiration and associated soil physicochemical properties in the tropical deciduous forests of Bhawal National Park, Bangladesh. Significant differences in soil respiration among *Shorea robusta* , *Albizia procera* , and *Acacia auriculiformis* indicate that species‐specific belowground processes influence forest carbon dynamics. *Acacia auriculiformis* exhibited the highest soil respiration, whereas *A. procera* maintained greater soil organic carbon and total nitrogen, suggesting contrasting patterns of carbon turnover and soil carbon storage among the studied species.

Among the measured soil properties, moisture content and exchangeable calcium were identified as the strongest predictors of soil respiration, highlighting their importance in regulating belowground carbon dynamics. The negative relationship between soil respiration and soil organic carbon further suggests that variation in carbon turnover may not be explained solely by carbon quantity but may also reflect differences in carbon stabilization or accessibility.

The contrasting responses of *A. procera* and *A. auriculiformis* , despite both belonging to the Fabaceae, indicate that species‐specific functional traits are more likely to regulate rhizosphere carbon dynamics than phylogenetic affinity or species origin alone. These findings suggest that forest restoration and afforestation programmes should consider species‐specific belowground functional characteristics, together with aboveground productivity, when selecting tree species for sustainable management.

Although this study provides important insights, litter characteristics, root functional traits, microbial communities, and seasonal variation were not evaluated. Future studies integrating these factors with long‐term field monitoring will improve understanding of species‐specific controls on soil carbon dynamics and support evidence‐based restoration of tropical deciduous forests.

## Funding

This research was funded by the Centre for Advanced Studies and Research in Biological Sciences, University of Dhaka, Bangladesh.

## Ethics Statement

The authors have nothing to report.

## Conflicts of Interest

The authors declare no conflicts of interest.

## Supporting information


**Table S1:** Soil physicochemical properties of rhizosphere soils at two depths (Upper: 0–15 cm, Lower: 15–30 cm) for three dominant plant species in Bhawal National Park (BNP).
**Table S2:** Results of Analysis of Variance (ANOVA) showing the effects of different plant species, soil depths, and their interactions on soil physicochemical properties.
**Table S3:** PCA (Principal Component Analysis) results of soil physicochemical properties significantly correlated with Soil respiration.
**Table S4:** Stepwise Multiple Linear Regression Subset Summary.
**Figure S1:** Canonical Discriminant Analysis (CDA) biplot showing the relationships among soil physicochemical properties in the rhizosphere soils of three plant species: *Acacia auriculiformis* , *Albizia procera* , and *Shorea robusta* . The vectors represent soil properties, including pH (soil pH), moisture content (MC), conductivity, BD (bulk density), OC (organic carbon), TN (total nitrogen), TP (total phosphorus), and the concentrations of Ca, Mg, K, sand, silt, and clay and their influence on the canonical discriminant functions. Clusters represent the distinct soil property profiles associated with each plant species.

## Data Availability

The data that supports the findings of this study are openly available in figshare at http://doi.org/10.6084/m9.figshare.31441099.

## References

[pei370200-bib-0001] Alam, M. A. , M. Yeasin , and A. Ahmed . 2024. “Carbon Pool and Respiration of Rhizosphere Soils of Different Mangrove Plant Species in Bangladesh Sundarbans.” Bangladesh Journal of Botany 53, no. 1: 131–140. 10.3329/bjb.v53i1.72257.

[pei370200-bib-0002] Alauddin, M. , M. N. Hossain , M. B. Islam , S. Islam , and M. K. Islam . 2020. “Management Strategies for Sustainable Forest Biodiversity Conservation in Protected Areas of Bangladesh: A Study of Bhawal National Park, Gazipur.” Grassroots Journal of Natural Resources 3, no. 3: 56–72. 10.33002/nr2581.6853.03035.

[pei370200-bib-0003] Allen, S. E. , H. M. Grimshaw , J. A. Parkinson , and C. Quarnby . 1974. Chemical Analysis of Ecological Materials. Blackwell Science Inc.

[pei370200-bib-0004] Binkley, D. , and C. Giardina . 1998. “Why Do Tree Species Affect Soils? The Warp and Woof of Tree‐Soil Interactions.” Biogeochemistry 42: 89–106. 10.1023/A:1005948126251.

[pei370200-bib-0005] Bond‐Lamberty, B. , V. Bailey , M. Chen , C. Gough , and R. Vargas . 2018. “Globally Rising Soil Heterotrophic Respiration Over Recent Decades.” Nature 560, no. 7716: 80–83. 10.1038/s41586-018-0358-x. 30068952

[pei370200-bib-0006] Bond‐Lamberty, B. , A. Ballantyne , E. Berryman , et al. 2024. “Twenty Years of Progress, Challenges, and Opportunities in Measuring and Understanding Soil Respiration.” Journal of Geophysical Research: Biogeosciences 129, no. 2: e2023JG007637. 10.1029/2023JG007637.

[pei370200-bib-0007] Bronick, C. J. , and R. Lal . 2005. “Soil Structure and Management: A Review.” Geoderma 124, no. 1–2: 3–22. 10.1016/j.geoderma.2004.03.005.

[pei370200-bib-0008] Chaturvedi, M. , S. Lakshmi , D. Kumar , and J. Gupta . 2025. “Carbon Cycling and Climate Feedback in Terrestrial Ecosystems.” In Green Equilibrium: Deciphering Earth's Ecosystems for a Sustainable Tomorrow, 97–117. Springer Nature Singapore. 10.1007/978-981-96-3993-9_5.

[pei370200-bib-0009] Das, P. P. , K. R. Singh , G. Nagpure , et al. 2022. “Plant‐Soil‐Microbes: A Tripartite Interaction for Nutrient Acquisition and Better Plant Growth for Sustainable Agricultural Practices.” Environmental Research 214: 113821. 10.1016/j.envres.2022.113821. 35810815

[pei370200-bib-0011] Fan, S. Q. , S. L. Peng , and B. M. Chen . 2024. “A Global Meta‐Analysis Reveals the Positive Effect of Invasive Alien Plants on Soil Heterotrophic Respiration.” Soil Biology and Biochemistry 194: 109450. 10.1016/j.soilbio.2024.109450.

[pei370200-bib-0012] Forrester, D. I. , J. Bauhus , A. L. Cowie , and J. K. Vanclay . 2006. “Mixed‐Species Plantations of Eucalyptus With Nitrogen‐Fixing Trees: A Review.” Forest Ecology and Management 233: 211–230. 10.1016/j.foreco.2006.05.012.

[pei370200-bib-0013] Fu, R. , X. Xu , Y. Yu , Y. Zhang , Z. Sun , and X. Tao . 2021. “Forest Soil Respiration Response to Increasing Nitrogen Deposition Along an Urban‐Rural Gradient.” Global Ecology and Conservation 27: e01575. 10.1016/j.gecco.2021.e01575.

[pei370200-bib-0014] Gan, D. , J. Feng , M. Han , H. Zeng , and B. Zhu . 2021. “Rhizosphere Effects of Woody Plants on Soil Biogeochemical Processes: A Meta‐Analysis.” Soil Biology and Biochemistry 160: 108310. 10.1016/j.soilbio.2021.108310.

[pei370200-bib-0015] Guo, Y. , Z. Zeng , J. Wang , J. Zou , Z. Shi , and S. Chen . 2023. “Research Advances in Mechanisms of Climate Change Impacts on Soil Organic Carbon Dynamics.” Environmental Research Letters 18, no. 10: 103005. 10.1088/1748-9326/acfa12.

[pei370200-bib-0016] Gupta, S. R. , G. W. Sileshi , R. K. Chaturvedi , and J. C. Dagar . 2023. “Soil Biodiversity and Litter Decomposition in Agroforestry Systems of the Tropical Regions of Asia and Africa.” In Agroforestry for Sustainable Intensification of Agriculture in Asia and Africa, edited by J. C. Dagar , S. R. Gupta , and G. W. Sileshi , 515–568. Springer Nature Singapore. 10.1007/978-981-19-4602-8_16.

[pei370200-bib-0017] Haney, R. L. , W. F. Brinton , and E. Evans . 2008. “Soil CO_2_ Respiration: Comparison of Chemical Titration, CO_2_ IRGA Analysis and the Solvita Gel System.” Renewable Agriculture and Food Systems 23, no. 2: 171–176. 10.1017/S174217050800224X.

[pei370200-bib-0018] Hao, Y. , J. Mao , C. M. Bachmann , et al. 2025. “Soil Moisture Controls Over Carbon Sequestration and Greenhouse Gas Emissions: A Review.” NPJ Climate and Atmospheric Science 8, no. 1: 16. 10.1038/s41612-024-00888-8.

[pei370200-bib-0019] Huang, N. , L. Gu , and Z. Niu . 2014. “Estimating Soil Respiration Using Spatial Data Products: A Case Study in a Deciduous Broadleaf Forest in the Midwest USA.” Journal of Geophysical Research: Atmospheres 119, no. 11: 6393–6408. 10.1002/2013JD020515.

[pei370200-bib-0020] Huang, Z. , Y. Liu , P. Huang , Z. Li , and X. Zhang . 2023. “A New Concept for Modelling the Moisture Dependence of Heterotrophic Soil Respiration.” Soil Biology and Biochemistry 185: 109147. 10.1016/j.soilbio.2023.109147.

[pei370200-bib-0021] Huq, S. I. , and M. D. Alam . 2005. A Handbook on Analyses of Soil, Plant and Water. BACER‐DU, University of Dhaka.

[pei370200-bib-0022] Hurteau, M. D. 2021. “The Role of Forests in the Carbon Cycle and in Climate Change.” In Climate Change, edited by T. M. Letcher , 561–579. Elsevier. 10.1016/B978-0-12-821575-3.00027-X.

[pei370200-bib-0023] Islam, S. S. , M. I. Ali , M. A. Haydar , et al. 2017. “Background Gamma Radiation Mapping in Forest Ecosystem of Bangladesh: A Study on the Radioactivity Distribution in the National Reserve Forest of Gazipur.” Radiation Protection and Environment 40, no. 2: 73–83. 10.4103/rpe.RPE_17_17.

[pei370200-bib-0024] Jaafar, S. M. , F. Metali , and R. S. Sukri . 2022. “Acacia Invasion Differentially Impacts Soil Properties of Two Contrasting Tropical Lowland Forests in Brunei Darussalam.” Journal of Tropical Ecology 38, no. 5: 259–266. 10.1017/S0266467422000141.

[pei370200-bib-0025] Jaafar, S. M. , and R. S. Sukri . 2023. “Data on the Physicochemical Characteristics and Texture Classification of Soil in Bornean Tropical Heath Forests Affected by Exotic *Acacia mangium* .” Data in Brief 51: 109670. 10.1016/j.dib.2023.109670. 37869617 PMC10587726

[pei370200-bib-0026] Jin, C. , H. Lei , J. Chen , et al. 2023. “Effect of Soil Aeration and Root Morphology on Yield Under Aerated Irrigation.” Agronomy 13, no. 2: 369. 10.3390/agronomy13020369.

[pei370200-bib-0027] Jobbágy, E. G. , and R. B. Jackson . 2001. “The Distribution of Soil Nutrients With Depth: Global Patterns and the Imprint of Plants.” Biogeochemistry 53, no. 1: 51–77. 10.1023/A:1010760720215.

[pei370200-bib-0028] Kabir, D. S. , and A. Z. Ahmed . 2005. “Wildlife Biodiversity in Bhawal National Park: Management Techniques and Drawbacks of Wildlife Management and Nature Conservation.” Our Nature 3, no. 1: 83–90. 10.3126/on.v3i1.340.

[pei370200-bib-0029] Khanikar, C. , S. S. S. ingh , and S. I. Bhuyan . 2023. “Physico‐Chemical Characteristics of Rhizospheric Soils of Some Important Medicinal Plants Found in North East India.” Advances in Zoology and Botany 11, no. 3: 194–202. 10.13189/azb.2023.110305.

[pei370200-bib-0030] Kruse, J. , J. Simon , and H. Rennenberg . 2013. “Soil Respiration and Soil Organic Matter Decomposition in Response to Climate Change.” Developments in Environmental Science 13: 131–149. 10.1016/B978-0-08-098349-3.00007-4.

[pei370200-bib-0031] Lal, R. 2005. “Forest Soils and Carbon Sequestration.” Forest Ecology and Management 220, no. 1–3: 242–258. 10.1016/j.foreco.2005.08.015.

[pei370200-bib-0032] Lee, Y. K. , and S. Y. Woo . 2012. “Changes in Litter, Decomposition, Nitrogen Mineralization and Microclimate in *Acacia mangium* and *Acacia auriculiformis* Plantation in Mount Makiling, Philippines.” International Journal of Physical Sciences 7, no. 12: 1976–1985.

[pei370200-bib-0033] Lin, Q. , Q. Wu , C. Chen , et al. 2025. “Species‐Specific Effects of Litter Management on Soil Respiration Dynamics in Urban Green Spaces: Implications for Carbon Cycling and Climate Regulation.” Forests 16, no. 4: 642. 10.3390/f16040642.

[pei370200-bib-0034] Lone, P. A. , J. A. Dar , K. Subashree , et al. 2019. “Impact of Plant Invasion on Physical, Chemical and Biological Aspects of Ecosystems: A Review.” Tropical Plant Research 6, no. 3: 528–544. 10.22271/tpr.2019.v6.i3.067.

[pei370200-bib-0035] Mondol, M. A. , M. A. Wadud , and G. M. Rahman . 2010. “Effect of Deforestation on Physicochemical Characteristics of Soil of Bhawal and Madhupur National Park Range of Sal Forest.” Journal of Agroforestry and Environment 5, no. 1: 143–148.

[pei370200-bib-0036] Moreno Roblero, M. D. , J. Pineda Pineda , M. T. Colinas León , and J. Sahagún Castellanos . 2020. “Oxygen in the Root Zone and Its Effect on Plants.” Revista Mexicana de Ciencias Agrícolas 11, no. 4: 931–943. 10.29312/remexca.v11i4.2128.

[pei370200-bib-0037] Mukul, S. A. , M. A. Arfin‐Khan , and M. B. Uddin . 2021. “Invasive Alien Species of Bangladesh.” In Invasive Alien Species: Observations and Issues From Around the World, edited by T. Pullaiah and M. R. Ielmini , 1–5. Wiley Online Library. 10.1002/9781119607045.ch13.

[pei370200-bib-0038] Okalebo, J. R. , K. W. Gathua , and P. L. Woomer . 1993. Laboratory Methods of Soil and Plant Analysis: A Working Manual. Tropical Soil Biology and Biofertility Programme.

[pei370200-bib-0039] Ontong, N. , R. Poolsiri , S. Diloksumpun , D. Staporn , and M. Jenke . 2023. “Effects of Tree Functional Traits on Soil Respiration in Tropical Forest Plantations.” Forests 14, no. 4: 715. 10.3390/f14040715.

[pei370200-bib-0040] Patra, A. , V. K. Sharma , D. J. Nath , et al. 2021. “Impact of Soil Acidity Influenced by Long‐Term Integrated Use of Enriched Compost, Biofertilizers, and Fertilizer on Soil Microbial Activity and Biomass in Rice Under Acidic Soil.” Journal of Soil Science and Plant Nutrition 21, no. 1: 756–767. 10.1007/s42729-020-00398-5.

[pei370200-bib-0041] Phillips, C. L. , B. Bond‐Lamberty , A. R. Desai , et al. 2017. “The Value of Soil Respiration Measurements for Interpreting and Modeling Terrestrial Carbon Cycling.” Plant and Soil 413, no. 1: 1–25. 10.1007/s11104-016-3084-x.

[pei370200-bib-0042] Pisani, O. , K. M. Hills , D. Courtier‐Murias , M. L. Haddix , E. A. Paul , and R. T. Conant . 2014. “Accumulation of Aliphatic Compounds in Soil With Increasing Mean Annual Temperature.” Organic Geochemistry 76: 118–127. 10.1016/j.orggeochem.2014.07.009.

[pei370200-bib-0043] Propa, M. J. , M. I. Hossain , and A. Ahmed . 2021. “Carbon Stock and Respiration of Rhizosphere Soils of Sal ( *Shorea robusta* Roxb. Ex. Gaertn. F.) in Relation to Some Environmental Variables of Different Sal Forest Stands of Bangladesh.” Bangladesh Journal of Botany 50, no. 3: 685–693. 10.3329/bjb.v50i3.55849.

[pei370200-bib-0044] Qi, Q. , D. Zhang , M. Zhang , S. Tong , W. Wang , and Y. An . 2021. “Spatial Distribution of Soil Organic Carbon and Total Nitrogen in Disturbed *Carex* Tussock Wetland.” Ecological Indicators 120: 106930. 10.1016/j.ecolind.2020.106930.

[pei370200-bib-0045] Rahman, M. , A. Nishat , and H. Vacik . 2009. “Anthropogenic Disturbances and Plant Diversity of the Madhupur Sal Forests ( *Shorea robusta* C.F. Gaertn) of Bangladesh.” International Journal of Biodiversity Science & Management 5, no. 3: 162–173. 10.1080/17451590903236741.

[pei370200-bib-0046] Rahman, M. U. , T. Dey , and J. Biswas . 2023. “Land‐Use Change and Forest Cover Depletion in Bhawal National Park, Gazipur, Bangladesh From 2005 to 2020.” Environmental Monitoring and Assessment 195, no. 1: 201. 10.1007/s10661-022-10764-8. 36525097

[pei370200-bib-0047] Roychand, P. , and P. Marschner . 2015. “Effects of Different Rates of Ca2+ Addition on Respiration and Sorption of Water‐Extractable Organic C to a Vertisol Subsoil.” Communications in Soil Science and Plant Analysis 46, no. 2: 185–194. 10.1080/00103624.2014.967858.

[pei370200-bib-0048] Sarkar, S. , D. K. Das , A. Singh , et al. 2024. “Seasonal Variations in Soil Characteristics Control Microbial Respiration and Carbon Use Under Tree Plantations in the Middle Gangetic Region.” Heliyon 10: e11673. 10.1016/j.heliyon.2024.e35593. PMC11379560 39247289

[pei370200-bib-0049] Sebastian, K. , M. Monika , T. R. Przemyslaw , L. Wojciech , and M. Jacek . 2023. “Soil Organic Carbon and Mineral Nitrogen Contents in Soils as Affected by Their pH, Texture, and Fertilization.” Agronomy 13, no. 1: 267. 10.3390/agronomy13010267.

[pei370200-bib-0050] Sharma, U. C. , M. Datta , and V. Sharma . 2025. “Chemistry, Microbiology, and Behaviour of Acid Soils.” In Soil Acidity: Management Options for Higher Crop Productivity, 121–322. Springer Nature Switzerland. 10.1007/978-3-031-76357-1_3.

[pei370200-bib-0051] Si, P. , W. Shao , H. Yu , et al. 2018. “Rhizosphere Microenvironments of Eight Common Deciduous Fruit Trees Were Shaped by Microbes in Northern China.” Frontiers in Microbiology 9: 3147. 10.3389/fmicb.2018.03147. 30619213 PMC6305578

[pei370200-bib-0052] Sivaranjani, S. , M. S. Sarkar , V. P. Panwar , R. Pandey , A. P. Mishra , and U. Rathnayake . 2025. “Modeling Soil Respiration: Seasonal Variability and Drivers in Pine and Broad‐Leaved Forests of the Lower Himalayas.” Trees, Forests and People 20: 100804. 10.1016/j.tfp.2025.100804.

[pei370200-bib-0053] Sun, H. , L. Wang , A. Kumar , et al. 2025. “Nutrient Availability Mediates Organic Carbon Turnover in Paddy Soils Through Regulating Microbial Metabolism.” Geoderma 458: 117313. 10.1016/j.geoderma.2025.117313.

[pei370200-bib-0054] Tamura, M. , and V. Suseela . 2021. “Warming and Labile Substrate Addition Alter Enzyme Activities and Composition of Soil Organic Carbon.” Frontiers in Forests and Global Change 4: 691302. 10.3389/ffgc.2021.691302.

[pei370200-bib-0055] Velmourougane, K. , G. Saxena , and R. Prasanna . 2017. “Plant‐Microbe Interactions in the Rhizosphere: Mechanisms and Their Ecological Benefits.” In Plant‐Microbe Interactions in Agro‐Ecological Perspectives, edited by D. Singh , H. Singh , and R. Prabha , 193–219. Springer Singapore. 10.1007/978-981-10-6593-4_7.

[pei370200-bib-0056] Walkley, A. , and I. A. Black . 1934. “An Examination of the Degtjareff Method for Determining Soil Organic Matter, and a Proposed Modification of the Chromic Acid Titration Method.” Soil Science 37, no. 1: 29–38.

[pei370200-bib-0057] Wang, R. , Y. Han , Z. Meng , Y. Gao , and Z. Wu . 2024. “Soil Physicochemical Properties and Carbon Storage Reserve Distribution Characteristics of Plantation Restoration in a Coal Mining Area.” Forests 15, no. 11: 1876. 10.3390/f15111876.

[pei370200-bib-0058] Wuyts, K. , A. De Schrijver , J. Staelens , and K. Verheyen . 2013. “Edge Effects on Soil Acidification in Forests on Sandy Soils Under High Deposition Load.” Water, Air, and Soil Pollution 224, no. 6: 1598. 10.1007/s11270-013-1545-x.

[pei370200-bib-0059] Xia, Y. , J. Feng , H. Zhang , et al. 2024. “Effects of Soil pH on the Growth, Soil Nutrient Composition, and Rhizosphere Microbiome of *Ageratina adenophora* .” PeerJ 12: e17231. 10.7717/peerj.17231. 38646477 PMC11027909

[pei370200-bib-0060] Xu, S. , K. Li , G. Li , et al. 2022. “Canada Goldenrod Invasion Regulates the Effects of Soil Moisture on Soil Respiration.” International Journal of Environmental Research and Public Health 19, no. 23: 15446. 10.3390/ijerph192315446. 36497518 PMC9741181

[pei370200-bib-0061] Yao, Y. , Q. Dai , R. Gao , X. Yi , Y. Wang , and Z. Hu . 2023. “Characteristics and Factors Influencing Soil Organic Carbon Composition by Vegetation Type in Spoil Heaps.” Frontiers in Plant Science 14: 1240217. 10.3389/fpls.2023.1240217. 37900766 PMC10602739

