# Supplementary material for: Species‐Specific Controls on Rhizosphere Soil Respiration in a Tropical Deciduous Forest of Bangladesh
Source: Plant Environ Interact. 2026 Aug 9;7(4):e70200. doi: 10.1002/pei3.70200 (PMC13454544; doi:10.1002/pei3.70200)
Supplement: Supplementary file 1 [file PEI3-7-e70200-s001.docx]

Table S1: Soil physicochemical properties of rhizosphere soils at two depths (Upper: 0–15 cm, Lower: 15–30 cm) for three dominant plant species in Bhawal National Park (BNP).

| Plant | *Acacia auriculiformis* | | *Albizia procera* | | *Shorea robusta* | |
| --- | --- | --- | --- | --- | --- | --- |
| Depth | Upper | Lower | Upper | Lower | Upper | Lower |
| pH | 5.21 ± 0.14 (a) | 5.37± 0.21(a) | 4.75± 0.07(b) | 4.86± 0.15(b) | 4.73± 0.05(b) | 4.89± 0.13(b) |
| MC (%) | 22.96 ± 0.65 (ab) | 25.07 ± 0.62 (ab) | 20.78± 0.33 (b) | 22.86± 0.84 (ab) | 23.6 ± 1.94 (b) | 26.19 ± 2.09 (a) |
| OC (%) | 0.58± 0.19 (bc) | 0.41± 0.17 (c) | 1.08± 0.23 (a) | 0.71± 0.03 (b) | 0.74± 0.07 (b) | 0.59 ± 0.07 (bc) |
| Rs (mg CO_2_/g soil | 59.82± 1.65 (ab) | 61.69± 2.15 (a) | 54.04± 2.19 (c) | 57± 0.67 (bc) | 57.33± 1.27 (b) | 59.16± 1.28 (ab) |
| Conductivity (µS/cm) | 113.67± 9.23 (a) | 124.5 ± 10 (a) | 97.17± 8.08 (a) | 159.33± 10.26 (a) | 118.9± 21.09 (a) | 143.95± 38.85 (a) |
| TN (%) | 0.07± 0.006 (cd) | 0.05± 0.009 (d) | 0.16± 0.02 (a) | 0.11± 0.008 (bc) | 0.11± 0.01 (b) | 0.09± 0.017 (bc) |
| TP (%) | 0.1± 0.02 (a) | 0.09± 0.01 (a) | 0.11± 0.02 (a) | 0.08± 0.01 (a) | 0.12± 0.03 (a) | 0.09± 0.02 (a) |
| Sand (%) | 51.67± 2.08 (a) | 44 ± 1 (bc) | 42.44± 1.66 (c) | 37.34± 2.08 (c) | 48.66± 2.93 (ab) | 39.29± 3.2 (c) |
| Silt (%) | 29.67± 0.57 (a) | 24.67± 4.16 (ab) | 18.58± 1.53 (bc) | 22.62± 2.02 (bc) | 18.9± 2.18 (c) | 19.66 ± 2.85 (bc) |
| Clay (%) | 18.67± 1.52 (c) | 31.33± 4.5 (b) | 38.95± 2.87 (a) | 40.33± 0.57 (a) | 32.88± 1.61 (b) | 41.12 ± 2.39 (a) |
| Ca (meq. /100 g) | 4.69 ± 0.03 (ab) | 4.9 ± 0.07 (a) | 3.98 ± 0.09 (d) | 4.49 ± 0.31 (bc) | 4.29 ± 0.03 (c) | 4.49 ± 0.06 (b) |
| Mg (meq/100g) | 1.3 ± 0.05 (abc) | 1.37 ± 0.02 (a) | 1.12 ± 0.03 (d) | 1.26 ± 0.08 (bc) | 1.23 ± 0.02 (c) | 1.3 ± 0.03 (ab) |
| K (ppm) | 63.47± 12.84 (a) | 70.18± 16.25 (a) | 61.23 ± 5.07 (a) | 88.22 ± 21.42 (a) | 72.83 ± 18.45 (a) | 70.34 ± 18.97 (a) |

Groups sharing the same letter are not significantly different from each other.

Table S2: Results of Analysis of Variance (ANOVA) showing the effects of different plant species, soil depths, and their interactions on soil physicochemical properties.

| **pH** | **Source** | **DF** | **Adj SS** | **Adj MS** | **F-Value** | **P-Value** |
| --- | --- | --- | --- | --- | --- | --- |
|  | Plant | 2 | 1.13584 | 0.567922 | 37.46 | 0.000 |
|  | Depth | 1 | 0.12398 | 0.123976 | 8.18 | 0.008 |
|  | Plant*Depth | 2 | 0.00368 | 0.001838 | 0.12 | 0.886 |
|  |  |  |  |  |  |  |
| **MC** | **Source** | **DF** | **Adj SS** | **Adj MS** | **F-Value** | **P-Value** |
|  | Plant | 2 | 43.792 | 21.8959 | 7.41 | 0.003 |
|  | Depth | 1 | 30.017 | 30.0169 | 10.16 | 0.004 |
|  | Plant*Depth | 2 | 0.481 | 0.2406 | 0.08 | 0.922 |
|  |  |  |  |  |  |  |
| **OC** | **Source** | **DF** | **Adj SS** | **Adj MS** | **F-Value** | **P-Value** |
|  | Plant | 2 | 0.49009 | 0.24505 | 18.15 | 0.000 |
|  | Depth | 1 | 0.29766 | 0.29766 | 22.04 | 0.000 |
|  | Plant*Depth | 2 | 0.05694 | 0.02847 | 2.11 | 0.142 |
|  |  |  |  |  |  |  |
| **Rs**  **(mg CO_2_/ g soil)** | **Source** | **DF** | **Adj SS** | **Adj MS** | **F-Value** | **P-Value** |
|  | Plant | 2 | 82.230 | 41.1152 | 19.52 | 0.000 |
|  | Depth | 1 | 28.990 | 28.9904 | 13.76 | 0.001 |
|  | Plant*Depth | 2 | 1.553 | 0.7764 | 0.37 | 0.695 |
|  |  |  |  |  |  |  |
| **Conductivity** | **Source** | **DF** | **Adj SS** | **Adj MS** | **F-Value** | **P-Value** |
|  | Plant | 2 | 703.5 | 351.8 | 0.50 | 0.612 |
|  | Depth | 1 | 6269.9 | 6269.9 | 8.91 | 0.006 |
|  | Plant*Depth | 2 | 2222.2 | 1111.1 | 1.58 | 0.225 |

Table S2: Continued

| **TN** | **Source** | **DF** | **Adj SS** | **Adj MS** | **F-Value** | **P-Value** |
| --- | --- | --- | --- | --- | --- | --- |
|  | Plant | 2 | 0.015643 | 0.007821 | 30.43 | 0.000 |
|  | Depth | 1 | 0.004878 | 0.004878 | 18.98 | 0.000 |
|  | Plant*Depth | 2 | 0.000947 | 0.000474 | 1.84 | 0.179 |
|  |  |  |  |  |  |  |
| **TP** | **Source** | **DF** | **Adj SS** | **Adj MS** | **F-Value** | **P-Value** |
|  | Plant | 2 | 0.000801 | 0.000400 | 0.69 | 0.512 |
|  | Depth | 1 | 0.002841 | 0.002841 | 4.87 | 0.036 |
|  | Plant*Depth | 2 | 0.000201 | 0.000100 | 0.17 | 0.843 |
|  |  |  |  |  |  |  |
| **Sand** | **Source** | **DF** | **Adj SS** | **Adj MS** | **F-Value** | **P-Value** |
|  | Plant | 2 | 189.55 | 94.776 | 12.63 | 0.000 |
|  | Depth | 1 | 319.70 | 319.702 | 42.62 | 0.000 |
|  | Plant*Depth | 2 | 21.71 | 10.855 | 1.45 | 0.254 |
|  |  |  |  |  |  |  |
| **Silt** | **Source** | **DF** | **Adj SS** | **Adj MS** | **F-Value** | **P-Value** |
|  | Plant | 2 | 288.362 | 144.181 | 22.77 | 0.000 |
|  | Depth | 1 | 0.027 | 0.027 | 0.00 | 0.948 |
|  | Plant*Depth | 2 | 64.109 | 32.054 | 5.06 | 0.014 |
|  |  |  |  |  |  |  |
| **Clay** | **Source** | **DF** | **Adj SS** | **Adj MS** | **F-Value** | **P-Value** |
|  | Plant | 2 | 807.22 | 403.611 | 76.21 | 0.000 |
|  | Depth | 1 | 324.15 | 324.155 | 61.21 | 0.000 |
|  | Plant*Depth | 2 | 98.19 | 49.097 | 9.27 | 0.001 |
|  |  |  |  |  |  |  |
| **Ca** | **Source** | **DF** | **Adj SS** | **Adj MS** | **F-Value** | **P-Value** |
|  | Plant | 2 | 1.0546 | 0.52728 | 51.38 | 0.000 |
|  | Depth | 1 | 0.5596 | 0.55964 | 54.53 | 0.000 |
|  | Plant*Depth | 2 | 0.1119 | 0.05594 | 5.45 | 0.011 |
|  |  |  |  |  |  |  |
| **Mg** | **Source** | **DF** | **Adj SS** | **Adj MS** | **F-Value** | **P-Value** |
|  | Plant | 2 | 0.064838 | 0.032419 | 22.19 | 0.000 |
|  | Depth | 1 | 0.052829 | 0.052829 | 36.16 | 0.000 |
|  | Plant*Depth | 2 | 0.006757 | 0.003379 | 2.31 | 0.119 |
|  |  |  |  |  |  |  |
| **K** | **Source** | **DF** | **Adj SS** | **Adj MS** | **F-Value** | **P-Value** |
|  | Plant | 2 | 192.5 | 96.24 | 0.31 | 0.738 |
|  | Depth | 1 | 634.9 | 634.87 | 2.03 | 0.166 |
|  | Plant*Depth | 2 | 1009.1 | 504.54 | 1.61 | 0.219 |

Table S3: PCA (Principal Component Analysis) results of soil physicochemical properties significantly correlated with Soil respiration

| **Variable** | **PC1** | **PC2** | **Correlation with Rs** |
| --- | --- | --- | --- |
| Eigenvalue | 4.521 | 1.18 |  |
| Cumulative percentage (%) | 64.6 % | 81.5 % |  |
| pH | 0.385 | -0.361 | 0.50** |
| MC | 0.216 | 0.697 | 0.61*** |
| OC | -0.416 | -0.187 | -0.67*** |
| TN | -0.430 | -0.076 | -0.72*** |
| Silt | 0.300 | -0.551 | 0.36* |
| Ca | 0.442 | -0.070 | 0.70*** |
| Mg | 0.402 | 0.186 | 0.66*** |

Table S4: Stepwise Multiple Linear Regression Subset Summary

| Model | Unstandardized coefficient | | t-value | Significance |
| --- | --- | --- | --- | --- |
|  | β | β Standard error |  |  |
| Intercept | 24.7132 | 4.7246 | 5.231 | 0.000 *** |
| MC | 0.4299 | 0.1182 | 3.637 | 0.000 *** |
| Ca | 5.2066 | 1.0866 | 4.792 | 0.000 *** |

**Fig. S1.** Canonical Discriminant Analysis (CDA) biplot showing the relationships among soil physicochemical properties in the rhizosphere soils of three plant species: Acacia auriculiformis, Albizia procera, and Shorea robusta. The vectors represent soil properties, including pH (soil pH), moisture content (MC), conductivity, BD (bulk density), OC (organic carbon), TN (total nitrogen), TP (total phosphorus), and the concentrations of Ca, Mg, K, sand, silt, and clay and their influence on the canonical discriminant functions. Clusters represent the distinct soil property profiles associated with each plant species.
